# Union Meeting of the Connecticut Valley Dental Society, the New England Dental Society, and the Connecticut State Dental Association, at Springfield, Mass., October 23, 24, and 25, 1889

**Published:** 1890-05

**Authors:** 


### UNION MEETING OF THE CONNECTICUT VALLEY DENTAL SOCIETY, THE NEW ENGLAND DENTAL SOCIETY, AND THE CONNECTICUT STATE DENTAL ASSOCIATION, AT SPRINGFIELD, MASS., OCTOBER 23, 24, and 25, 1889.[1]

(Continued from page 235.)

### DENTAL EDUCATION.

BY N. MORGAN, D.D.S., SPRINGFIELD, MASS.

Much has been and is constantly being written on the subject of dental education. Is the profession as a whole even reasonably well educated? A few have been fortunate in acquiring an advanced professional education, and are qualified to associate with

---

[1] Reported for the International Dental Journal by Geo. A. Maxfield, D.D.S., Holyoke, Mass.

members of any learned profession, and many of these men are endeavoring to lift the laggards into the light and, after revealing to them their need, to hold up high standards of attainment. That which was the peculiar privilege of the few may become, to a large degree, the possession of many. Are we satisfied with the sordid element in our nature which looks only to the return of dollars for our work? or, shall we cultivate the humanitarian element which is a blessing to the sufferers who come to us for relief?

To claim that wonderful advancement has not been made would be libellous; dentistry is moving forward, but we are not satisfied with the return for the efforts put forth,—it is not all that could be desired or reasonably expected.

If this statement is accepted as correct there must be some reason for our present condition. Would it not be well to try to analyze our situation to some extent? Perhaps we may arrive at more efficient methods for its improvement.

First let us glance at our present educational facilities,—their advantages and failures. For those who can avail themselves of them,—and every individual who now enters the profession should do so,—there are the dental colleges with their corps of earnest and efficient workers. They furnish facilities for the acquisition of education, both of mind and the equally essential one of hands skilled to work out the suggestions of the mind. That the colleges are to be praised for such efficient work we all agree. At the same time they do not claim to educate a student so that he will have no further need of increasing fitness for work, but rather an adaptation of the individual for additional study and research.

A second class to consider is made up of many practitioners who either could not or would not take advantage of these privileges. These have, however, the same educational facilities at hand common to us all. I refer to the dental literature which includes some journals of great excellence; dental societies, clubs, etc. These organizations are usually open to all who have a desire for improvement, at the same time being of slight expense, compared with most social clubs.

This, then, seems to be our condition as a profession; there are a multitude of men of various ages,—many with slight early educational advantages and at present without the perception of *how* to study, or the ability, so to speak, of knowing how to set themselves to work in a practical and productive way. "But," remarks some one, "we have known all this for years, and have we not been striving to bring about a more desirable condition? and are we not almost discouraged in the attempt to make our profession an honored one, and to place it on a plane with the medical profession, aspiring to have our vocation recognized by the world as a branch of the healing art?" What is the result? We are forced to confess ourselves still in bonds to our surroundings, but not in the way of permitting them to cause discouragement. We have not the history of the medical profession, it is true, but we are capable of *making* history. To-day we are not so proud of our ancestry as our deeds. It is the apostolic "forgetting that which is behind and reaching out to that which is before" which is to make our personal or professional success. Retrospection may be productive of good, if our mistakes and failures lead us to change our ways so as to avoid the evils of the past.

We believe that there is a still better outlook, if not through one avenue, perhaps another will be opened. Unforeseen exceptional advancement may be made in the coming year or years, and the object of presenting this paper is to offer possible suggestions in the way of its achievement.

For a long time we have been convinced that our present desultory method should give place to a systematic course of study outside the college walls. We read many essays and articles, good enough in themselves, but without collateral study they lose much of their power. How different would have been the effect of Professors Andrews's and Sudduth's lectures of last evening if the hearers had previously read something relative to the subject? Some, if they study at all, prefer *special lines* of study, which in our opinion should *follow* a course having a general outlook. One apparent need in this direction is an element which shall draw us together, a uniting of interests, "a brotherhood," if you choose to call it so. Cannot the "Chautauqua" system be so modified as to meet our demands? Most of us are somewhat familiar with the workings of that system, and know of the wonderful educational and moral influence it has and is yet exerting. There are now circles in almost every civilized portion of the world, over one hundred thousand persons having been allied to the movement. This result has not come by chance but by natural laws.

President Garfield, in a speech at Chautauqua, said, "It has been the struggle of the world to get more leisure, but it was left to Chautauqua to show how to use it;" the real Chautauqua idea being the saving of time, the gathering up the moments which are ordinarily wasted, and so employing them as to bring rich returns. The student is encouraged to study because, virtually, he has a tutor; because a well-defined course of study is prepared for him; because a multitude of associates are pursuing the same, and a feeling of sympathy is common among them.

Besides the study, there are the "Local Circles," which hold regular meetings in the interests of their work, whereby great enthusiasm is aroused. At the end of each year comes a test to be answered in writing, and to those who persevere and pass successful examinations for four years a diploma is awarded. After this general course, special courses may be pursued, which are increasingly interesting and instructive and may be continued indefinitely.

The query now is, Can we not adapt to our own need a system similar to this? If we have not already such text-books as we need, can they not be prepared,—books concisely written and suited to such a purpose? We have sometimes overlooked the best in our lack of time to discriminate carefully, and the inferior on which we often stumble discourages us from perusing further, but we may have a systematic pursuit of such knowledge as will in some way or degree fit us to become more of a power in every feature of our profession. Is not that method which "reaches down and lifts men up" one by which we may bring the greatest number into line? The greatest difficulty in leading men into the student life is the first step; for after the taste is formed the difficulties begin to vanish.

A thirst for knowledge is a law of our being given for good of self and that of the whole world. If the desire is dormant it may be revived by suitable influences. Our particular branch of science is as distinguished as any for the demand it makes upon skill in manipulation, and although the laboratory and chair furnish the best school for our handicraft, yet we need as much knowledge as we can assimilate and organize into a basis for our work,—not thinking of our own pleasure alone, but as an obligation to those who put themselves under our care.

If such courses of study were presented to our profession to-day, the needed requisites being at hand, how many would rejoice and take advantage of the privilege? How many *here present* would be glad of the opportunity? How many would form clubs or "circles" of two, six, a dozen, or more, meeting from time to time for discussions of subjects gone over, for experiments, or anything which would help to make study a pleasure and our life brighter and better worth the living.

I am sure members of the old "Springfield Dental Club" will agree with me that pleasure and friendly interests are promoted and the weariness of many an hour lightened by such intercourse. How much stronger the bond of sympathy if there were clubs in Boston, Hartford, Providence, and every other place where a club could be organized, all using the same course of study,—or some perhaps taking the advance courses, but all in the same general organization. Some will make excuses,—want of time, want of opportunity, want of talent or of cleverness. Are we making the best use of all our time? Are we improving the one or more talents given us? Are we as clever as we might be if we sought to know what another's cleverness has done? Great and good thoughts may lie in the mind of an individual brother for his personal good; he may not be able to write an acceptable article for a journal, but meeting another who possesses the gift of clear and forcible speech without the power of originality, they can conjointly benefit the brotherhood. There is a vast amount of thinking that ought to be in the market, bringing in great returns. It might surprise us to learn that while on change, our idea, expressed faultily though it may have been, was taken possession of by one who so transformed it with beauty and strength that when it returns to us and others, it may aid us in our work, or, warming and refining our hearts, help us to be more perfect in character and life.

How such an organization can be brought about is hardly possible for one single-handed to determine. Perhaps a committee might bring the subject before the American Association.

Some journal might be published in its interests. Possibly Professor Sudduth and the managers of the INTERNATIONAL JOURNAL might use its pages for the purpose, and the enterprise be managed by them and an associate committee from the American Association combined, the courses of study and text-books to be selected and arranged under their supervision. It would be necessary for every person to become a regular matriculant and pay the nominal fee therefor, in order to enjoy the privileges of such an organization and become eligible to receive a diploma at graduation. The matriculation fee should be as low as possible and books furnished at reasonable rates. The whole expense need not be so great as to prevent any one from entering the ranks. Probably there are few among us who do not squander more each year than would cover the necessary expense for the course.

Moreover, the increased respect in which each member would be held by the community would tend to increase and improve his practice, thus bringing a material return for all investment in the cause.

It is to be emphatically stated that this method is in no sense suggested to take the place of the present college course, but is subsidiary to it. Rather is it to *precede* and *follow* the college course, and for those already in the profession who are debarred from its advantages. As long as we live we may learn if we will.

DISCUSSION.

*Dr. Barrett.*—The subject is one that should appeal to the inmost professional heart of every dentist who loves his calling. What has the future in store for us? Are we to sit still, to rest content with what we are, or shall we make an advance that will be commensurate with our past history? This matter is not now presented for the first time. The West has had it under consideration, and Dr. J. D. Moody, of Illinois, has written some very thoughtful papers in advocacy of a system of post-graduate study, somewhat upon the Chautauqua plan, which commends itself to every thoughtful dentist. In this new and aggressive department of medicine there is a hope found nowhere else. We are opening up new fields. We are free and untrammelled, and are not held down by the bonds of precedent and established custom, as are all the old professions. We have not to look back and guide our footsteps by the past, but need gaze only ahead. There are no narrow prejudices to hamper us, no old traditions to keep inviolate, but we are like children upon the virgin prairie, and may stray where we will. Divinity is held down by iron creeds which forbid free thought, and which prescribe a narrow path beyond which the members of that profession may not stray, nor even look. Law is more a matter of precedent than of prescribed rule, and courts in their interpretation, even of written statutes, are governed by the opinions of those who have gone before, and into the merits of which they may not inquire. Medicine is largely a system of empiricism, and it is necessarily the most conservative of professions. Consider what has been the fate of those in that profession who have advanced new theories and ideas. Think how the grand discoveries of Harvey and Jenner were received; remember the inertia of medicine and its intolerance of new principles, and then ask yourselves what hope the world of science has there. But we have no creeds, no precedents, no past by which we must be governed. Dentistry has such a chance as has never been granted the older professions, which have existed since man emerged from a state of barbarism and have not had our opportunities to start on a career guided only by nineteenth-century enlightenment. There is nothing to hinder an advance such as the world has never seen. Shall we make a record for the future, or shall we be content to rest where we are, thinking ourselves as large as we ever can be.

I have never seen a December in which I did not think I could view a manifest advance from the preceding January. All my life I have been climbing, climbing, standing on tiptoe, watching for the dawn of a yet earlier sun. I hope to continue thus until I shall for the last time hail the dawning of a new professional advancement. Dentistry is very largely made up of men who are necessarily earnestly looking towards the future, because they have no past to rest content in. It is in this that its hope lies. What shall the future bring forth?

Our colleges are continually taking advanced ground. But they do not yet represent the highest type of thought. They are not sufficiently ahead of the body of the profession. If they do not set a step that is sufficiently quick, if the rank and file are to tread upon their heels all the time, let the societies take the advance and give a new pace. We need a higher education, a deeper erudition. If this is not to be found in the schools, let the private offices furnish it. There is no reason why we should not blaze a path for the world through certain sections of the great forest of ignorance. There is an opportunity for dentistry to make for itself the very highest expression of scientific knowledge. All that is needful is that we should earnestly put our hands to the work and unitedly, shoulder to shoulder, take up the line of march for higher acquisitions. What could resist the impulse of a united profession, earnest for the right?

I am anxious to see the East take the lead in this great work. I want to see the sun of intelligence rise here and illumine the whole world. But this is not to be done by one man or one society. There must be united effort. The East and the West must be in accord, and all our societies must strike hands together. Therefore, I suggest co-operative committees from every society, which, acting together, shall thoroughly organize all earnest dentists into a compact body that shall lift a standard to which all must bow, and set a pace into which all the progressive men of the world must fall.

*Dr. W. H. Atkinson.*—The remarks we have just listened to coincide with what I think most about when I am alone. I have feared that we had come to a point in our ambition where we were about to take a permanent rest. It will be necessary to have some revival, at least of our ambition, to enable us practically to formulate and push forward the very desirable course suggested by the last speaker. I think his reference to the so-called professions being confined to iron creeds is fast being removed. For you scarcely find old fogies among old graduates of divinity or dentistry. They are not following out the old leads. Medicine especially has become so imbued with the spirit of progress under the hands of impatient young men that they have about discarded the old way of writing prescriptions. As far as divinity is concerned, you can scarcely find a man who has cheek enough to preach eternal hell any longer. As to the other profession, I do not feel there is much to encourage us, for they go by precedent. They esteem it a breach of brotherly feeling and respect to file a decision that has not been rendered before in some of the courts, irrespective of the testimony before the court, or of the facts of the case, and so they decide more frequently against truth and innocence. It looks foolish to have a set of boys—as we may call them—who have discovered a better way to instruct human beings, and learn from them how to deal with an adverse environment of the human mind. But we must go to them if we progress.

There is no such thing as getting a formulative syllabus, that will reveal to us all the pathological changes. I defy any man to so state the history of any case of disease as to make it cognate with any disease he or anybody else will ever see afterwards. Could we get a little more familiar with a few established basal principles, then we might have something like what I suggested at the last meeting of the American Dental Association.

The time to do something in the direction of the suggestions in the paper is now, when our minds are drawn to it and we see the necessity of it.

It will be the happiest day of my life when I can rise above my ignorance and prejudice and sincerely investigate a case, and it will intensify my joy if I could find my brethren following in that same journey.

*Dr. Miller.*—I feel that this is a subject of very great importance, and I hope some means will be taken to follow out the suggestions in the paper. It seems to me a committee should take this into further consideration and carry it forward and not let it drop here. I therefore move you, that a committee of three be appointed to bring in a list of five or more that shall be a committee to take charge of this subject and to report some plan—that will be practical—at some future meeting.

*Dr. Shepard.*—If any action is taken, it should be by the whole profession, not by a few societies. Now, if these societies could take the initiative, and appoint a committee which would bring other societies to work in the same direction, you will finally secure a congress that would act for the whole country.

*Dr. Rhein.*—I agree with Dr. Barrett's sentiment on this question, and I would like to amend the motion. It is not to give a course of instruction in any particular school. I therefore move the following amendment: That a committee of three be appointed to try and secure co-operation of various dental societies in the United States, to further some plan of this kind, and such committee to confer with similarly appointed committees from other societies and report at some future meeting.

*Dr. Maxfield.*—The suggestions made by Dr. Morgan in his paper are too good to be lost, and that they may take some practical shape, I would urge the original motion.

*Dr. McManus.*—A large committee will be better than the one suggested by Dr. Rhein. I think the chairman would like to be relieved, and therefore I think it might be well for the chairman to name a committee to report a committee for adoption by this meeting. I think the subject suggested is practical, and if carried out, future generations will rise and call the men of this day blessed.

The president then put the motion and it was carried, and Drs. Morgan, Brackett, and McManus were appointed.

After a conference, this committee reported the following names, which were adopted as the permanent committee to take this subject in charge: Dr. N. Morgan, of Springfield, Mass.; Dr. George A. Maxfield, of Holyoke, Mass.; Dr. William J. Rider, of Danbury, Conn.; Dr. George L. Parmele, of Hartford, Conn.; Dr. W. E. Page, of Boston, Mass.; Dr. A. M. Dudley, of Salem, Mass.

Subject passed.

(To be continued.)

